# A highly efficient Cu(In,Ga)(S,Se)_2_ photocathode without a hetero-materials overlayer for solar-hydrogen production

**DOI:** 10.1038/s41598-018-22827-3

**Published:** 2018-03-26

**Authors:** Byungwoo Kim, Gi-Soon Park, Sang Youn Chae, Min Kyu Kim, Hyung-Suk Oh, Yun Jeong Hwang, Woong Kim, Byoung Koun Min

**Affiliations:** 1 Clean Energy Research Center, Korea Institute of Science and Technology, Hwarang-ro 14-gil 5, Seongbuk-gu, Seoul, 02792 Republic of Korea; 2 Department of Materials Science and Engineering, Korea University, 145 Anam-ro, Seongbuk-gu, Seoul, 02841 Korea; 3 Green School, Korea University, 145 Anam-ro, Seongbuk-gu, Seoul, 02841 Republic of Korea

## Abstract

Surface modification of a Cu(In,Ga)(S,Se)_2_ (CIGSSe) absorber layer is commonly required to obtain high performance CIGSSe photocathodes. However, surface modifications can cause disadvantages such as optical loss, low stability, the use of toxic substances and an increase in complexity. In this work, we demonstrate that a double-graded bandgap structure (top-high, middle-low and bottom-high bandgaps) can achieve high performance in bare CIGSSe photocathodes without any surface modifications via a hetero-materials overlayer that have been fabricated in a cost-effective solution process. We used two kinds of CIGSSe film produced by different precursor solutions consisting of different solvents and binder materials, and both revealed a double-graded bandgap structure composed of an S-rich top layer, Ga- and S-poor middle layer and S- and Ga-rich bottom layer. The bare CIGSSe photocathode without surface modification exhibited a high photoelectrochemical activity of ~6 mA·cm^−2^ at 0 V *vs*. RHE and ~22 mA·cm^−2^ at −0.27 V *vs*. RHE, depending on the solution properties used in the CIGSSe film preparation. The incorporation of a Pt catalyst was found to further increase their PEC activity to ~26 mA·cm^−2^ at −0.16 V *vs*. RHE.

## Introduction

Solar hydrogen production is attracting attention as a fuel supply system due to its carbon free, environmentally friendly and sustainable characteristics. In such a system, a photoelectrode plays a very important role; its light absorption generates electron-hole pairs and its catalytic function decreases overpotential. Since the pioneering attempts with TiO_2_ material, many n-type semiconductor materials have been applied to and investigated for water oxidation photoelectrodes^1^. On the other hand, a p-type semiconductor material is also applicable to solar hydrogen production systems as a photocathode for proton reduction reaction. Various p-type materials such as metal oxides (Cu_2_O^2^, CuFeO_2_^3^, etc.) and III-V group materials (GaInN^4^, GaInP_2_^5^, etc.) have been considered as candidate materials for this reaction, but none has been very successful to date due to poor charge-carrier mobility, low stability, and high cost issues^6^. Therefore, developing a promising photocathode possessing outstanding characteristics at an effective cost is very important.

Among various p-type semiconductors, Cu(In,Ga)(S,Se)_2_ (CIGSSe) has recently received considerable attention due to its merits as an absorber for photocathodes such as high adsorption coefficient (~10^5^ cm^−1^)^7^, well-located conduction band edge and chemical stability^8^. For efficient photoelectrochemical (PEC) H_2_ evolution, the efficient separation of the photogenerated electron (e^−^) and hole (h^+^) is very important. To increase the separation ability, surface modifications with n-type hetero-materials are often applied to form the p-n junction at the interface with CIGSSe^9–19^. CdS has been widely used for this purpose because it forms a very harmonious p-n junction with CIGSSe^20^. For example, Domen and co-workers showed that the CdS modification of CuInGaSe_2_ photocathodes enhanced their photocurrent and onset potential for water splitting^9^. The calculated band alignment at the semiconductor-electrolyte interfaces for CuGaSe_2_ and CdS/CuGaSe_2_ indicated that the increased photocurrent of CdS/CuGaSe_2_ photocathode was produced by widening the depletion layer^10^. In addition, the diffusion of holes on the solid-electrolyte interface could be blocked as a result of CdS’s deeper (than CIGSSe’s) valence band maximum (VBM)^10,12^.

Due to these affirmative merits of surface modification with hetero-materials, the CdS/CIGSSe junction has long been successfully applied in thin film solar cell technologies. However, in the case of photocathode application of CIGSSe film for liquid-solid interfaces, the usage of n-type semiconductors for the surface modification is not highly necessary, unlike in solar cells. When a p-type semiconductor and electrolyte are in contact, hole flow occurs at the semiconductor-electrolyte interface to minimize the difference in the Gibbs energy of the semiconductor and electrolyte, and forming a depletion layer in the absence of a p-n junction^21^. In addition, the surface modification of CIGSSe using CdS has some critical drawbacks such as optical loss due to the relatively small bandgap (2.4 eV) of the CdS which partially overlaps with the visible light range^10–14^, the occurrence of e^−^/h^+^ recombination at defects of the CdS^19^, bad stability in aqueous media^12,15–18^, and toxicity of Cd, etc. Moreover, an additional process for CdS deposition demands cost enhancement for practical applications. However, a CIGSSe photocathode without n-type semiconductor material surface modification suffers from low PEC activity, presumably due to its low e^−^/h^+^ pair separation efficiency and e^−^/h^+^ recombination by holes diffused along the semiconductor/electrolyte interface.

Compositional grading in the CIGSSe absorber would solve these problems. The effects of compositional gradients are well documented in the solar cell field^22–25^. The relative amount of Ga and S determines the CIGSSe’s bandgap energy, which can range from 1.04 eV for pure CuInSe_2_ to 2.4 eV for pure CuGaS_2_. Higher Ga and S contents toward the back surface form a back surface field which assists free electrons generated outside the depletion layer to drift towards the depletion layer. Consequently, the separation ability of the absorber layer is enhanced without expanding the width of the depletion layer. In addition, the higher S content vicinity at the surface of the CIGSSe layer would lower the VBM, which repels the photogenerated holes away from the surface and prevents e^−^/h^+^ recombination^26^. Therefore, we can expect an effect similar to CdS modification through a compositional gradient without surface modification. However, little research has been done on the role of the compositional gradient in photocathode applications.

In this study, we demonstrate the possibility of the high hydrogen evolution reaction performance of only CIGSSe photocathodes without hetero-materials surface modification. Outstanding PEC properties could be achieved by a composition gradient within the CIGSSe absorber film associated with the double-graded bandgap property. Note that, in our study, a cost-effective solution process was used to synthesize CIGSSe thin films using two different precursor solutions in order to prepare CIGSSe films with different morphological characteristics (e.g. surface area, roughness and crystallinity). One precursor solution is based on a low viscosity polyvinyl acetate (PVA) binder and the other is based on a high viscosity ethyl cellulose (EC) binder^27,28^. The higher bandgap at the bottom was obtained due to the accumulation of Ga and S, while the higher bandgap at the surface was acquired due to surface sulfurization.

## Results and Discussion

### Morphology Analysis

**Figure 1:**
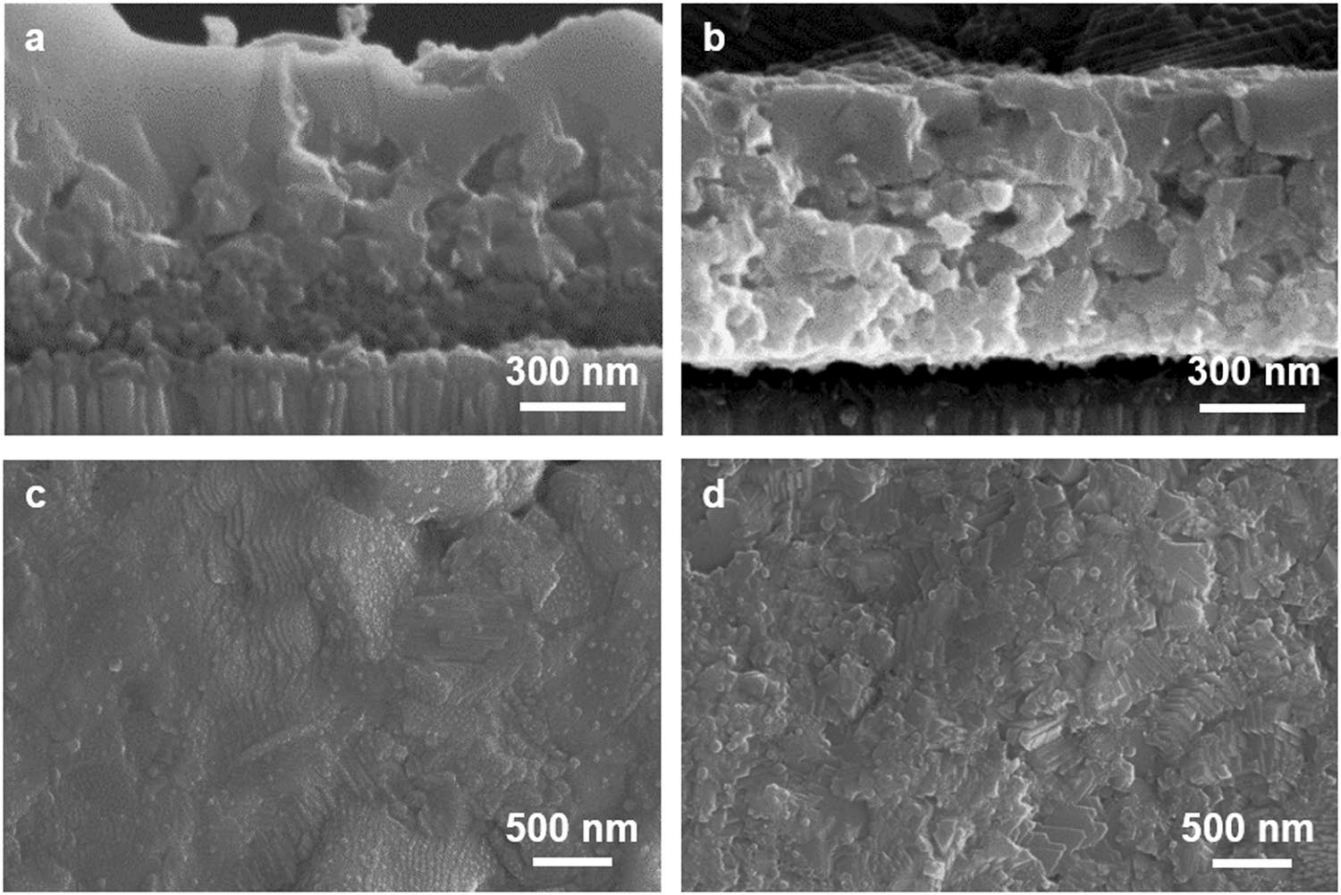
Cross-sectional (**a**,**b**) and top-view (**c**,**d**) SEM images of PVA-CIGSSe and EC-CIGSSe films: (**a**,**c**) PVA-CIGSSe (**b**,**d**) EC-CIGSSe.

In order to confirm the versatility of our process and induce different morphological characteristics, we synthesized CIGSSe absorber films (denoted as PVA-CIGSSe and EC-CIGSSe) by using two kinds of precursor solutions with different binders and solvents (see details in Experimental). The cross-sectional and surface SEM images of the CIGSSe films are shown in Fig. 1. Regardless of solution type, both CIGSSe films showed a similar bilayer structure with an upper large-grain layer and a lower fine-grain layer. Both films revealed a thickness of about 1 μm (Fig. 1a,b). These bilayers were due to the composition gradient and will be discussed in detail below. Although the structures of the two films were very similar, the upper part of the PVA-CIGSSe film showed slightly larger grain size and a flatter surface than the EC-CIGSSe film, which was confirmed by cross-sectional and surface images (Fig. 1a–d).

**Figure 2:**
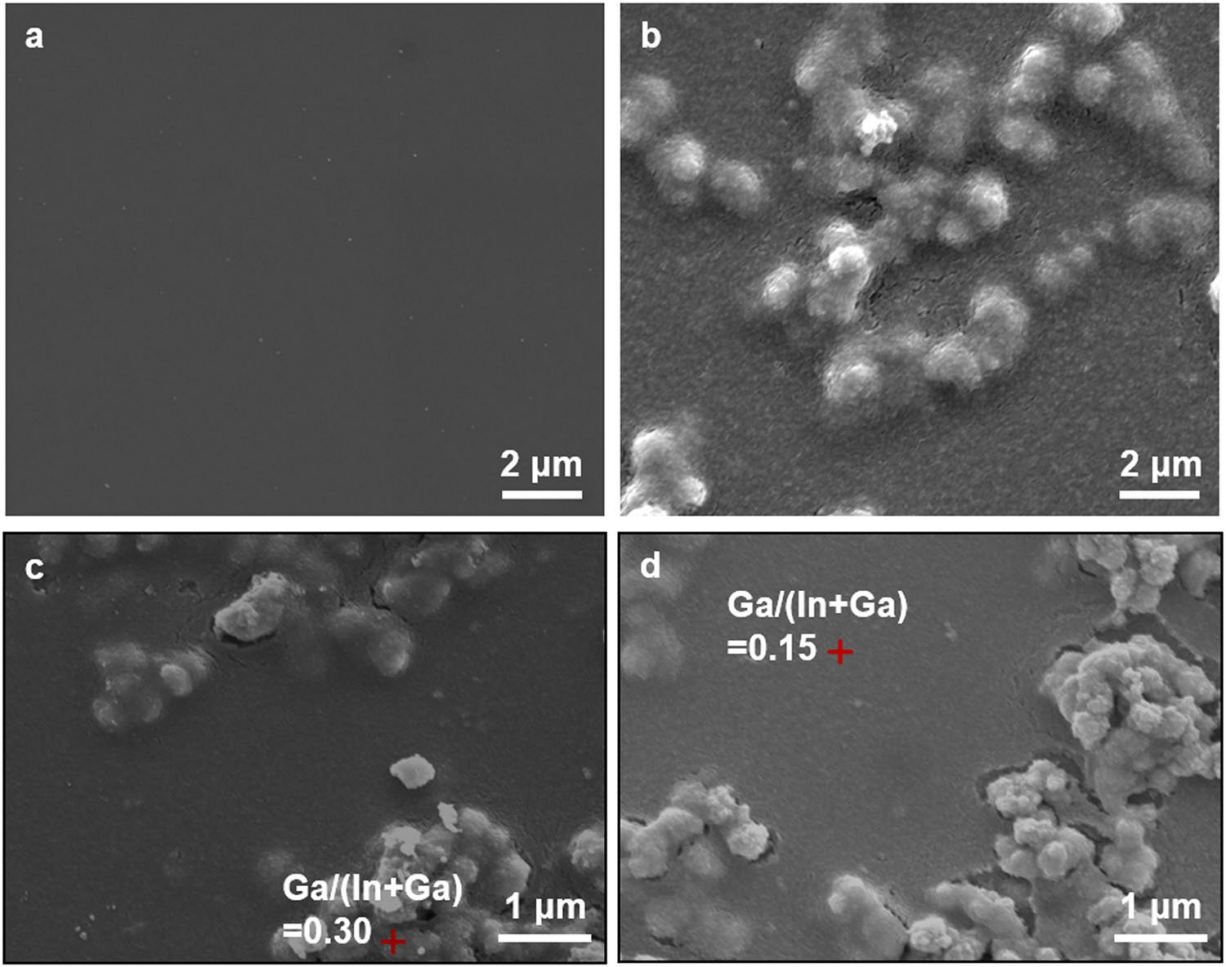
Top-view SEM images of (**a**) PVA-CIG and (**b**) EC-CIG films. (**c**,**d**) Agglomerated part (a red cross in c) and non-agglomerated part (a red cross in d) of an EC-CIG film. EDX was conducted at a red cross point.

To understand why the surface morphology of the two films was different, we investigated the surface of the films before the chalcogenization process (denoted as PVA-CIG and EC-CIG, Fig. 2). The PVA-CIG had a flat surface whereas the EC-CIG had irregularly agglomerated features in addition to the flat regions (Fig. 2a,b). EDX revealed that the agglomerated spots of the EC-CIG had a high Ga content (0.30) compared with the non-agglomerated spots (0.15) (Fig. 2c,d). After the chalcogenization process, the distinctive morphologies between the agglomerated and non-agglomerated regions disappeared, but the surface morphology of the CIGSSe films was significantly different, showing a much smoother surface for the PVA-CIGSSe than the EC-CIGSSE. This result indicates that solution properties influence the morphology of the intermediate state of the film, which also affects CIGSSe film quality.

**Figure 3:**
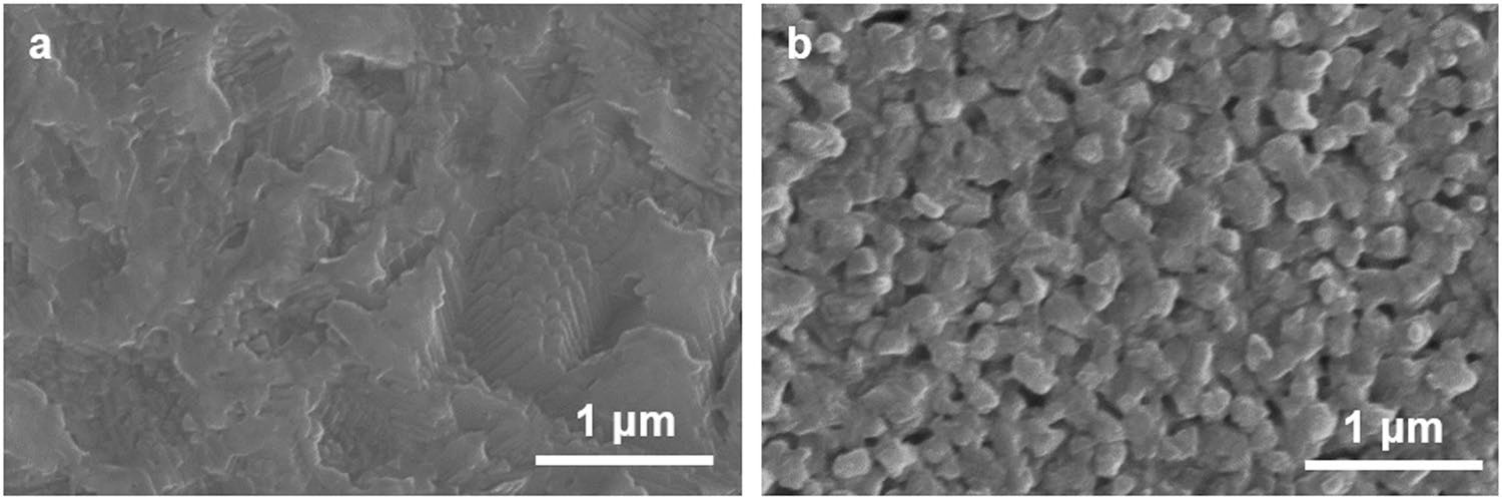
Top-view SEM images of one-less spin coated (**a**) PVA-CIGSSe and (**b**) EC-CIGSSe films.

The first example would be the surface area difference of both CIGSSe films estimated by AFM. The surface area is a very important parameter because it can directly influence the photocathode’s photocurrent generation. The two films showed different surface areas of 0.297 and 0.337 cm^2^ for PVA- CIGSSe and EC-CIGSSe electrodes, respectively. Note that the solution dependent film morphology changes were further confirmed by the synthesis of thinner CIGSSe films (~800 nm) by two solutions. A much denser and smoother surface morphology was observed in the PVA-CIGSSe, while a more coarse surface was seen in the EC-CIGSSe (Fig. 3).

### XRD Analysis

**Figure 4:**
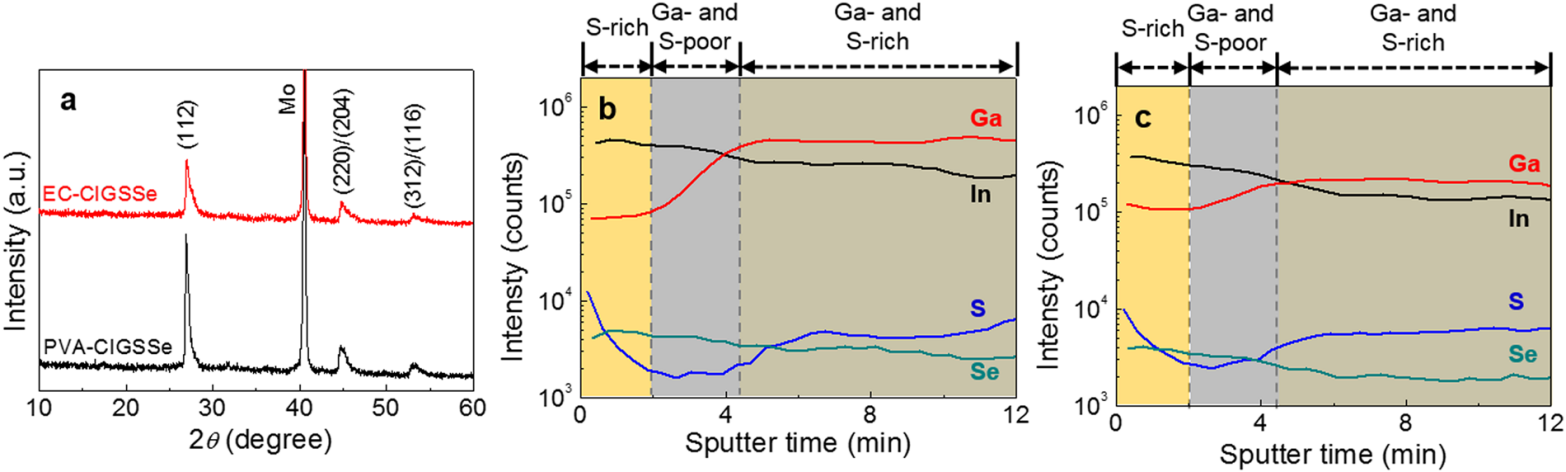
(**a**) XRD patterns of PVA-CIGSSe and EC-CIGSSe films and (**b**,**c**) the composition profile with respect to the (**b**) PVA-CIGSSe and (**c**) EC-CIGSSe films depth obtained by D-SIMS. (**a**, inset) The peak shape of (112) plane.

To investigate the crystal structure of the films, the XRD patterns of the PVA-CIGSSe and EC-CIGSSe were obtained (Fig. 4a). Both showed almost identical XRD patterns in good agreement with the typical CIGSSe chalcopyrite structure (JCPDS 35–1102). Binary compounds such as InSe and CuSe were not observed in the XRD patterns^29^. The Fig. 4a inset indicates the most intense peaks of the PVA-CIGSSe and EC-CIGSSe corresponding to (112) orientation. Both CIGSSe films exhibited an asymmetric shape widening toward a higher angle. According to our previous study, this asymmetric peak is attributed to the combination of a sharp peak Ga- and S-poor phase from the upper large-grain layer and a broad peak Ga- and S-rich phase from the bottom fine-grain layer^28^. On the other hand, PVA-CIGSSe exhibited higher peak intensity of (112) orientation than that of EC-CIGSSe, which indicates that higher crystallinity of PVA-CIGSSe may attributed to the more compact structure as confirmed in Fig. 3.

### Depth-dependent Composition Analysis

D-SIMS was carried out to analyze film depth-dependent composition variation, and the results showed three distinctive regions: S-rich top, Ga- and S-poor middle, and Ga- and S-rich bottom (Fig. 4b,c). The accumulation of Ga on the bottom layer commonly occurs in a sequential selenization process due to the favorable reaction kinetics of the lower activation energy of CuInSe_2_ phase (~124 KJ/mol) compared to CuInGaSe_2_ (~144 KJ/mol)^30^. Moreover, CuInSe_2_’s formation temperature is relatively lower than that of CuInGaSe_2_^31^. Since the bandgap increase is directly correlated with increases in Ga and S, the bottom layer should have a bandgap higher than the middle layer^22–24,32^. On the other hand, the layer near surface also has a higher bandgap due to the high content of S, which was achieved by continuing sulfurization even after selenization^33–36^. The increase in the S content at the bottom seems to be due to S preferentially reacting with Ga^37^. As a result, both CIGSSe films have a double-graded bandgap structure composed of top-high, middle-low and bottom-high bandgaps. The Fig. 4a inset shows that the 2*θ* value of the (112) peak of the PVA-CIGSSe was slightly lower than that of the EC-CIGSSe, indicating that the PVA-CIGSSe was incorporated with more In and/or Se rather than Ga and/or S in its crystal structure. Particularly, for the upper part of PVA-CIGSSe films, which may influence the XRD peak more, the composition analysis showed a higher concentration of In and Se than that of the EC-CIGSSe (Fig. 4b,c).

### Optical Properties

**Figure 5:**
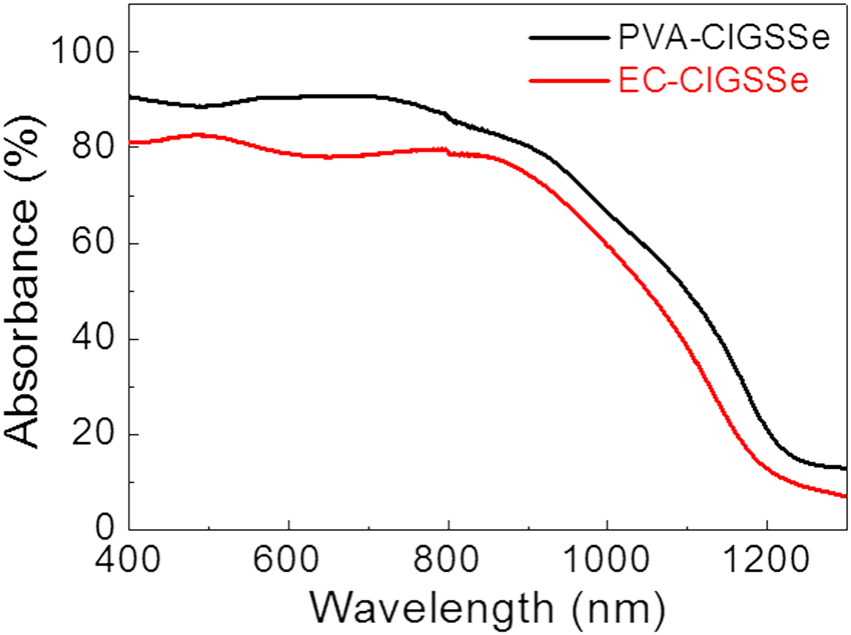
Absorption spectra of the PVA-CIGSSe film and the EC-CIGSSe film on glass substrate.

The optical properties of the CIGSSe films were investigated with UV-Vis spectroscopy. Absorbance was estimated through absorbance (%) = 100% -transmittance (%) - reflectance (%) using CIGSSe films separately prepared on a bare soda-lime glass substrate with an identical synthetic method to that prepared on a Mo-coated soda-lime glass substrate (Fig. 5)^38^. Although the EC-CIGSSe showed a slightly lower value than the PVA-CIGSSe, both films showed significant levels of absorbance in the short wavelength region (80~90% over 400 nm to 800 nm region). Notably, our CIGSSe films showed good absorbance at wavelengths shorter than 540 nm; therefore, they may lose this advantage when CdS is incorporated into a CIGSSe absorber due to its light absorption overlapping with that of the CdS (bandgap of 2.4 eV)^10–14^. The absorption edge wavelength of the PVA-CIGSSe was about 1220 nm, a value slightly longer than that of the EC-CIGSSe (1180 nm), and it implies that the bandgap of PVA-CIGSSe was slightly lower than that of EC-CIGSSe. The lower value of the PVA-CIGSSe bandgap may again be mainly due to the Ga-and S-poor phase of the upper part of the film, as confirmed by XRD and D-SIMS results. This implies that bandgap can also be affected by the type of paste solution, even though both precursor solutions had identical Cu/In/Ga ratios.

### PEC Characteristics

**Figure 6:**
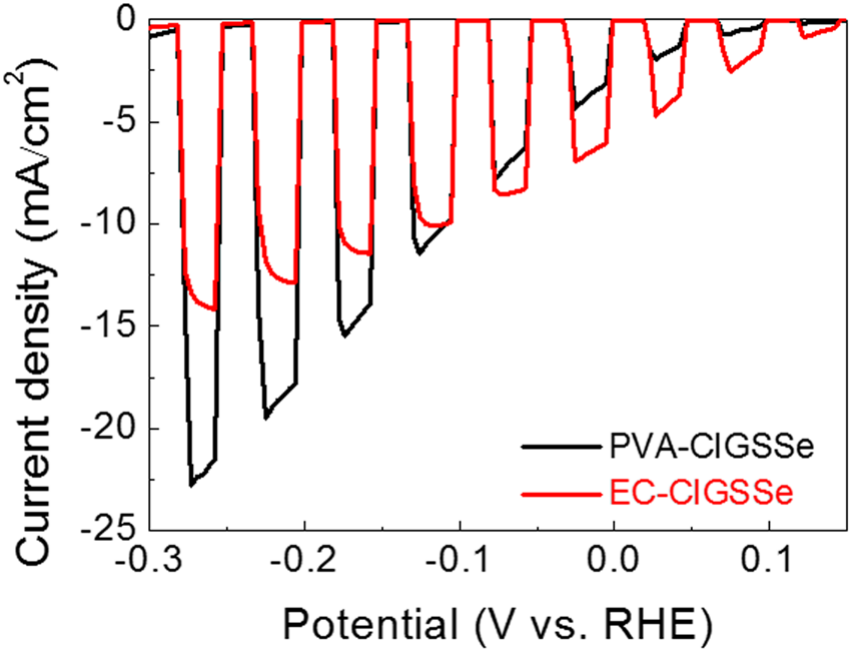
*I-V* curves of bare PVA-CIGSSe and EC-CIGSSe photocathodes.

Figure 6 shows the photocurrent generation behaviors of CIGSSe films. The photocurrent density of the PVA-CIGSSe and the EC-CIGSSe were approximately 3 mA·cm^−2^ and 6 mA·cm^−2^ at 0 V *vs*. RHE, and both CIGSSe films showed a considerable level of photocurrent density, even at low applied bias (0 V *vs*. RHE). In particular, the photocurrent density value of the EC-CIGSSe at 0 V *vs*. RHE is comparable with other reported solution-based CIGSSe photocathodes whose surfaces have been modified with hetero-materials overlayers (Table 1). The lower photocurrent density of the PVA-CIGSSe compared to EC-CIGSSe at low applied bias was presumably due to a more pronounced front bandgap grading profile attributed to the sharply increased S content on the surface. It can act as a barrier for electrons especially at low applied bias (near 0 V vs. RHE) because of the reduced SCR width^23^. While the EC-CIGSSe showed a higher photocurrent density compared to the PVA-CIGSSe at low applied bias (V > −0.1 V *vs*. RHE), the PVA-CIGSSe showed a higher photocurrent density at high applied bias (V < −0.1 V *vs*. RHE), mainly due to its lower grain boundary density and higher absorbance. The maximum photocurrent density of the PVA-CIGSSe was measured to 22 mA·cm^−2^.

**Table 1:** PEC Activity comparison of reported solution process chalcopyrite photocathodes for hydrogen evolution.

Photocathode	CIGSSe preparation method	Photocurrent at 0 *V*_RHE_ (mA·cm^−2^)	Maximum photocurrent (mA·cm^−2^)	Ref.
**PVA-CIGSxe**	**Spin coating**	**−3**	**−22 (at −0**.**27 V** ***vs***. **RHE)**	**This work**
**EC-CIGSSe**	**Spin coating**	**−6**	**−14 (at −0**.**25 V** ***vs***. **RHE)**	**This work**
**PVA-CIGSSe/Pt**	**Spin coating**	**−16**	**−26 (at −0**.**16 V** ***vs***. **RHE)**	**This work**
**EC-CIGSSe/Pt**	**Spin coating**	**−11**	**−24 (at −0**.**26 V** ***vs***. **RHE)**	**This work**
CIGSSe/ZnS/Pt	Spin coating	−16	−24 (at −0.3 V *vs*. RHE)	^43^
Bi:CIS_2_/CdS/ TiO_2_/Pt	Nanoparticle	−8	−8 (at 0 V *vs*. RHE)	^45^
CIGS_2_/CdS/Pt	Spin coating	−6	−11 (at ~−0.4 V *vs*. RHE)	^46^
CIS_2_/CdS/TiO_2_/Pt	Electrodeposition	−13	−14 (at −0.1 V *vs*. RHE)	^12^

**Figure 7:**
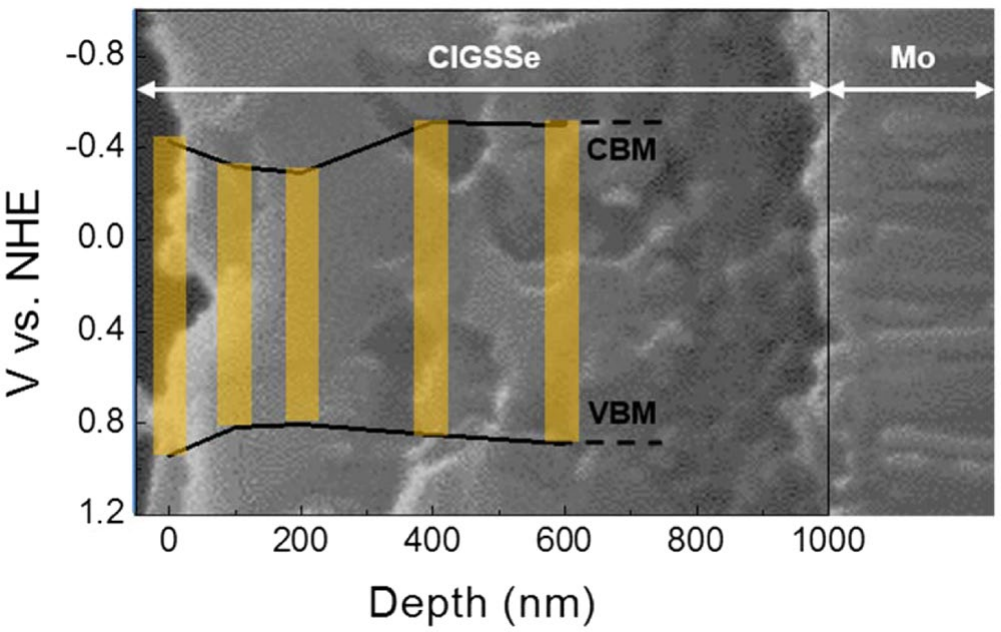
The distribution of CBM and VBM along with the PVA-CIGSSe film depth.

To gain further insight of outstanding properties of the bare CIGSSe photocathode even without surface modification, we conducted ultraviolet photoelectron spectroscopy (UPS) and Auger electron spectroscopy (AES) for PVA-CIGSSe to obtain VBM and bandgap as a function of depth (Figure S1 and S2). The range of the measured VBM in the bulk region of the film was found to be 0.8 ~ 0.9 V vs. NHE, which corresponds well to reported values^10,39^. The bandgap is determined based on the AES by the following equation^40^:

1 $${E}_{g}(x,y)=(1.00+0.13{x}^{2}+0.08{x}^{2}y+0.13xy+0.55\,x+0.54y)eV$$

where x and y are Ga/(Ga + In) and S/(Se + S), respectively. The CBM according to depth was obtained by adding the VBM and the bandgap. The band diagram of PVA-CIGSSe with respect to the depth was drawn as shown in Fig. 7. As illustrated, the back bandgap grading formed over a depth of about 200 to 400 nm almost exclusively changes only the CBM and does not change the VBM. Therefore, it forms additional electric field and helps the photogenerated minority carrier (electron) created outside the depletion layer migrate towards the depletion layer^22–25^. On the other hand, the front bandgap grading formed over a depth of about 0 to 200 nm which not only increases CBM but also lowers VBM, preventing surface state recombination by inhibiting photogenerated holes from migrating to electrolyte/CIGSSe interface^26^. In addition, surface sulfurization would passivate mid-gap recombination centers in the space charge region^36,41,42^. We have recently demonstrated that the surface state of CIGSSe can be passivated depending on sulfurization conditions, and thus increase photocurrent density^43^. For comparison ungraded films were prepared by applying the sulfurization process only while Se was being supplied in the chalcogenization process (see details in Supporting Information). PEC activity was obviously low in the ungraded film compared to that of the graded one as seen in Figure S3d. We also showed that double-graded bandgap structure CIGSSe films can have higher efficiency in solar cell performance compared to non-graded CIGSe films^28,44^.

**Figure 8:**
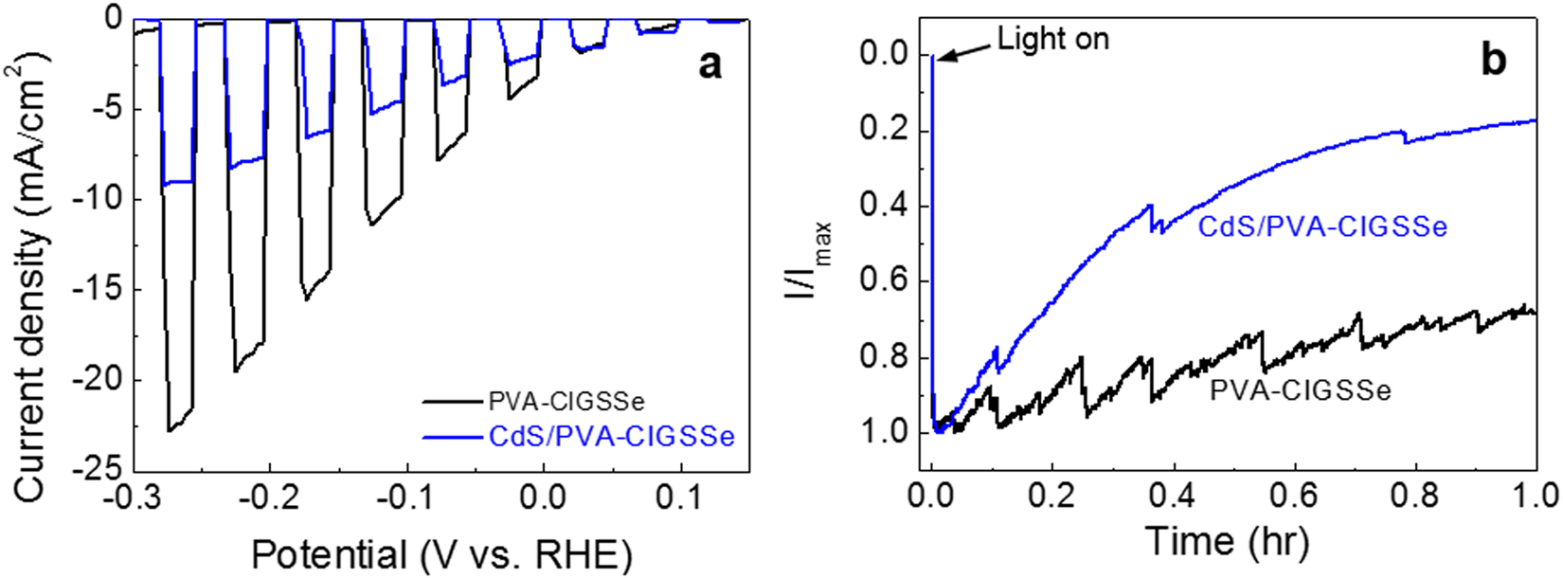
(**a**) *I-V* curves of bare PVA-CIGSSe and CdS/PVA-CIGSSe photocathodes. (**b**) *I-t* graph of PVA-CIGSSe and CdS/PVA-CIGSSe photocathodes under simulated sunlight at −0.2 V *vs*. RHE.

In order to confirm the possibility of further improvement of photocurrent density by surface modification with a hetero-materials layer, the PEC activity of a CdS-modified CIGSSe photocathode was also explored (Fig. 8a). A CdS-modified photocathode showed a decrease in photocurrent density compared to a PVA-CIGSSe. The photocurrent density of the CdS/PVA-CIGSSe was decreased from ~18 mA·cm^2^ to ~8 mA·cm^2^ at −0.2 V *vs*. RHE. In addition, the CdS modification decreased the durability of the CIGSSe photoelectrode (Fig. 8b). The photocurrent of the CdS/PVA-CIGSSe photocathode gradually diminished to only 18% of the initial photocurrent after 1 h while the photocurrent of the PVA-CIGSSe photocathode was maintained at 70%. Hydrogen production was confirmed by measuring hydrogen evolution rate with gas chromatography (Figure S4). Both photocurrent density and durability data show the negative effects of the CdS modification. The poor crystallinity of the CdS layer could generate numerous defects, which can result in serious charge recombination^19^. Moreover, it is well known that CdS used as a photocathode leads to self-photocorrosion from photogenerated holes^15,18^.

Our results imply that the negative impact of CdS modification is greater than the positive impact, *e*.*g*., widening the depletion layer and lowering the VBM. Moriya *et al*. showed that the CdS modification would expand the depletion layer of a CuGaSe_2_ photoelectrode from 250 nm to 320 nm under the supposition of a carrier concentration of 10^16^ cm^−3^ for both CuGaSe_2_ and CdS^10^. On the other hand, the bandgap grading of our PVA-CIGSSe films occurs over about 400 nm according to Fig. 7. In addition, surface sulfurization lowers VBM as mentioned above^26^.

To confirm the occurrence of self-photocorrosion, EDX was carried out on CdS/PVA-CIGSSe samples before and after durability testing. The Cd contents before and after durability testing were about 5.05 and 0.32%, respectively, clearly showing the degradation of CdS during the PEC reaction.

**Figure 9:**
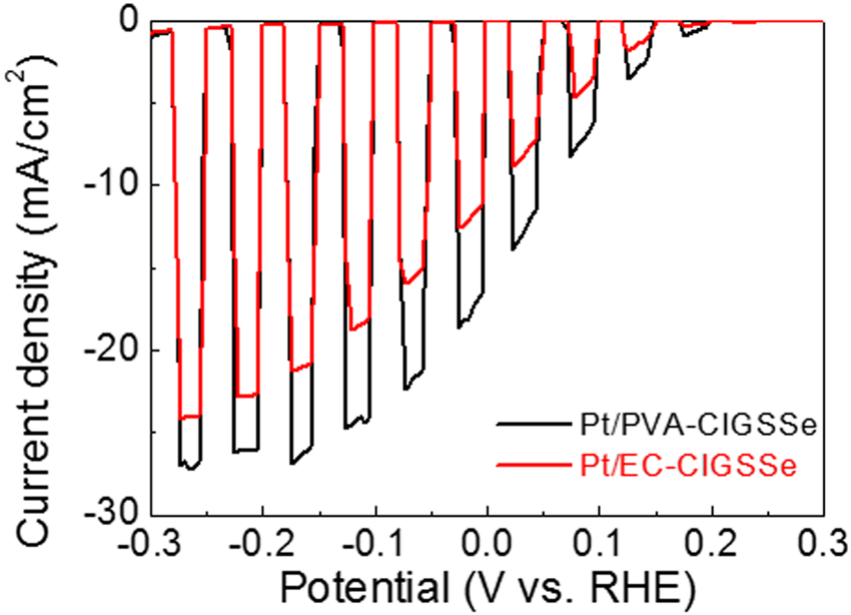
*I-V* curves of Pt deposited PVA-CIGSSe and EC-CIGSSe photocathodes.

The incorporation of a catalyst into CIGSSe film was also investigated (Fig. 9). A Pt catalyst was electrodeposited on the CIGSSe, and both CIGSSe films showed an increase in photocurrent density after the Pt deposition. The photocurrent densities of the PVA-CIGSSe and the EC-CIGSSe at 0 V *vs*. RHE improved from ~3 mA·cm^−2^ to ~16 mA·cm^−2^ and ~6 mA·cm^−2^ to ~11 mA·cm^−2^, respectively. Table 1 summarizes the photocurrent density at 0 V *vs*. RHE for previously reported CIGSSe photocathodes. The photocurrent density in the present study is higher than most of the CIGSSe photocathodes fabricated using solution processes and comparable to the most efficient solution-processed CIGSSe photocathodes (Table 1)^12,43,45,46^. The maximum photocurrent density of Pt-PVA/CIGSSe was about 26 mA·cm^−2^ at −0.16 V *vs*. RHE. To the best of our knowledge, this value is the highest efficiency among solution-processed CIGSSe films and comparable with vacuum-processed CIGSSe films^47–49^. Considering that relatively poor quality films are produced in the solution process, the PEC performance of these films is superior, indicating that the double-graded bandgap structure within the absorber film is very important in CIGSSe photocathode applications.

## Conclusion

In this work, the PEC properties of a double-graded bandgap bare CIGSSe absorber without surface modification were studied. Precursor solution processes using PVA in methanol and EC in ethanol solution were used. A double-graded bandgap structure was produced using the simple simultaneous sulfurization/selenization process. The bare EC-CIGSSe film yielded good PEC performance at low applied bias with a high photocurrent density of 6 mA·cm^−2^, while the PVA-CIGSSe film yielded a high maximum photocurrent density of 22 mA·cm^−2^ (−0.27 V *vs*. RHE). An n-type semiconductor (CdS) surface modification was found to be rather reduced in PEC activity. The use of Pt as a catalyst has been found to further increase the photocurrent density and onset potential of the films. After the Pt modification of the PVA-CIGSSe, a photocurrent density of 26 mA·cm^−2^ (−0.16 V *vs*. RHE) was obtained, the highest efficiency ever obtained by a solution-processed CIGSSe film. Our findings will probably be the cornerstone for a cost-effective CIGSSe photocathode.

## Methods

### Synthesis of the CIGSSe films

CIGSSe films based on a methanol solvent with a polyvinyl acetate (PVA) binder (denoted by PVA-CIGSSe) and an ethanol solvent with an ethyl cellulose (EC) + terpineol binder (denoted by EC-CIGSSe) films were prepared by a paste-coating method through multi-step heat treatment processes. A paste for PVA-CIGSSe films (PVA-paste) was prepared by dissolving Cu(NO_3_)_2_∙xH_2_O (99.999%, Sigma-Aldrich, 0.82 g), In(NO_3_)_3_∙xH_2_O (99.99%, Sigma-Aldrich, 1.12 g) and Ga(NO_3_)_3_∙xH_2_O (99.999%, Alfa Aesar, 0.41 g) in 8.4 mL of methanol (Daejung). PVA (M_W_ ~ 100,000, Sigma-Aldrich, 1 g) dissolved in 8.6 mL of methanol was mixed with the solution to control the viscosity during the spin coating. A paste for EC-CIGSSe films (EC-paste) was prepared by dissolving Cu(NO_3_)_2_∙xH_2_O 0.82 g, In(NO_3_)_3_∙xH_2_O 1.12 g and Ga(NO_3_)_3_∙xH_2_O 0.41 g in 5 mL of ethanol (Fischer). EC (48.0–49.5% (w/w) ethoxyl basis, Sigma-Aldrich, 0.5 g) dissolved in 10 mL of ethanol and 5 mL of terpineol was mixed with the solution.

The paste was spin cast onto a Mo-sputtered (500 nm) soda-lime glass substrate and dried on a hotplate at 340–350 °C for 30 min in a humidity-controlled atmosphere (<20%). Spin castings of the PVA- and EC-pastes were carried out under different conditions (2000 rpm, 40 sec for PVA-paste and 3000 rpm, 50 sec for EC-paste). The coating and drying processes were repeated six times for the PVA-paste and five times for the EC-paste to obtain the desired film thickness (~1 μm). To form the CIGSSe alloy, chalcogenization was carried out using a two-stage temperature controllable tube furnace. A selenium pellet (Se, Sigma-Aldrich, ~0.45 g) was placed in the tube furnace and heated to 550 °C to provide Se vapor. A flow of H_2_S gas (H_2_S(1%)/N_2_, 100 sccm) was provided during Se vapor evolution. The film was chalcogenized at 470 °C for 15 min. During chalcogenization, Se is supplied as vapor and reacts from the surface of the CuInGa metal precursor layer, CuInSe_2_ is generated from the surface, and Ga is pushed to the bottom of the film. Se is depleted during chalcogenization, and as Se is depleted H_2_S is the only reactant supplied to form graded S profile. The furnace was then cooled to room temperature with the flow of H_2_S gas. CdS modification (~60 nm) on the surface of the CIGSSe films was achieved via the chemical bath deposition (CBD) method^28^.

### PEC and electrochemical characterization

PEC and electrochemical properties were measured in an 0.5 M H_2_SO_4_ aqueous solution (pH ~0.6) using a potentiostat (Iviumstat) with a three electrode configuration. PVA-CIGSSe or EC-CIGSSe, a Pt coil, and an Ag/AgCl electrode (3 M NaCl) were used as a working electrode, a counter electrode and a reference electrode, respectively. In all cases, the active geometric area of the CIGSSe electrodes was determined to be 0.2826 cm^2^. The photocurrent density was measured by linear sweep voltammetry (LSV, 10∙mV s^−1^) under simulated sunlight (100 mW∙cm^−2^) from a solar simulator (Abet, Sun 2000) equipped with a 300 W xenon lamp and an AM 1.5 used as a light source, which is necessarily calibrated by a photovoltaic reference silicon solar cell (PV measurements, Inc.). The simulated sunlight was chopped during LSV measurement. Potentials on an RHE scale were calculated using the following formula: *V*_RHE_ = *V*_Ag/AgCl_ + 0.059 pH + 0.209 V. The Pt deposition was carried out in 1 mM H_2_PtCl_6_·6H_2_O (ACS reagent, ≥ 37.50% Pt basis, Sigma-Aldrich) at −0.15 V *vs*. Ag/AgCl for 360 sec. A durability test was conducted for 1 hr at −0.2 V *vs*. RHE under simulated sunlight conditions.

### Structural characterization

The structural and crystallinity characterization of the films was performed by scanning electron microscopy (SEM; FEI, Teneo Volume Scope and FEI, Inspect F) with an acceleration voltage of 15 kV and X-ray diffraction (XRD; Shimadzu, XRD-6000) with Cu Kα radiation (λ. = 0.15406 nm). Composition analysis was performed using energy-dispersive x-ray spectroscopy mounted on an SEM with an acceleration voltage of 20 kV (EDS; FEI, Teneo Volume Scope and FEI, Inspect F). The depth profiling was conducted by dynamic secondary ion mass spectrometry (D-SIMS; CAMECA, IMS4FE7). Optical properties were examined using an ultraviolet−visible−near-infrared (UV−vis−NIR) spectrophotometer (Varian, Cary 5000). The surface area was obtained by atomic force microscopy (AFM; Park Systems, XE-100).

## Electronic supplementary material

Supplementary Information
