# Supplementary material for: A highly efficient Cu(In,Ga)(S,Se)2 photocathode without a hetero-materials overlayer for solar-hydrogen production
Source: Sci Rep. 2018 Mar 26;8:5182. doi: 10.1038/s41598-018-22827-3 (PMC5980086; doi:10.1038/s41598-018-22827-3)
Supplement: Supplementary file 1 — Supplementary Information [file 41598_2018_22827_MOESM1_ESM.docx]

**SUPPORTING INFORMATION**

**A highly efficient Cu(In,Ga)(S,Se)2 photocathode without a hetero-materials overlayer for solar-hydrogen production**

Byungwoo Kim,^1,2^ Gi-Soon Park,^1,3^ Sang Youn Chae,^1^ Min Kyu Kim,^1,3^ Hyung-Suk Oh,^1^ Yun Jeong Hwang,^1^ Woong Kim^2*^ and Byoung Koun Min^1,3*^

^1^Clean Energy Research Center, Korea Institute of Science and Technology, Hwarang-ro 14-gil 5, Seongbuk-gu, Seoul 02792, Republic of Korea.

^2^Department of Materials Science and Engineering, Korea University, 145 Anam-ro, Seongbuk-gu, Seoul 02841, Korea.

^3^Green School, Korea University, 145 Anam-ro, Seongbuk-gu, Seoul 02841, Republic of Korea.

Corresponding Author

*E-mail: [bkmin@kist.re.kr](mailto:bkmin@kist.re.kr). Phone: +82 2 958 5853. Fax: +82 2 958 5809.

*E-mail: [woongkim@korea.ac.kr](mailto:woongkim@korea.ac.kr). Phone: +82 2 3290 3266.

**Supplementary Figures**


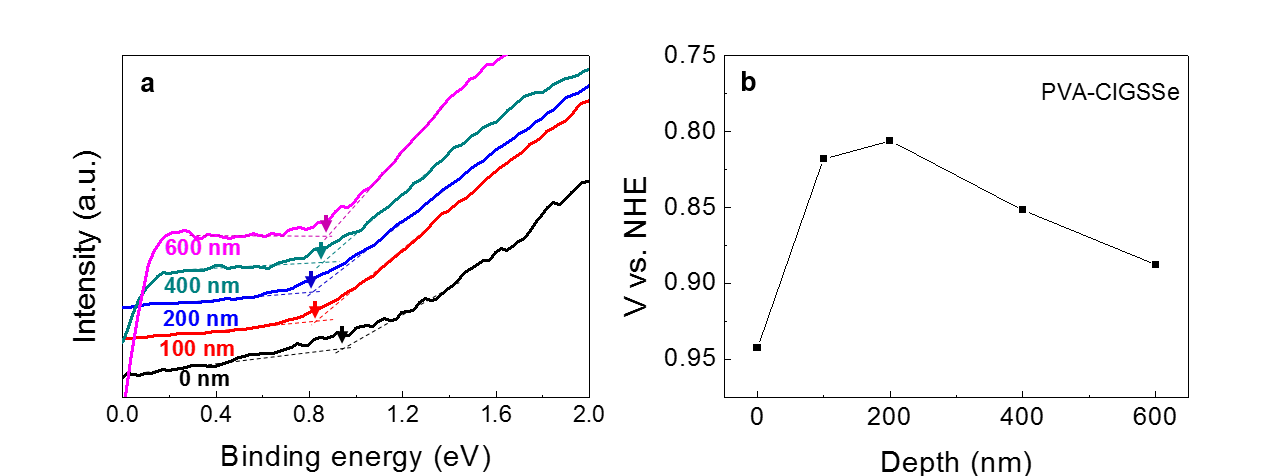


Figure S1. (a) Ultraviolet photoelectron spectroscopy (UPS) and (b) determined valence band maximum with respect to the PVA-CIGSSe film depth as derived from the UPS.


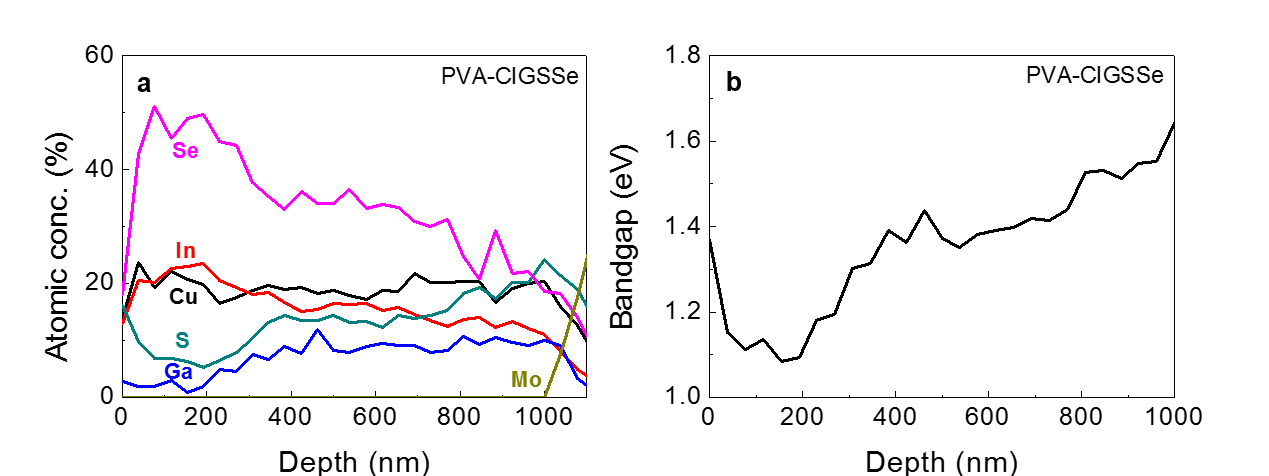


Figure S2. (a) Auger electron spectroscopy (AES) depth profiles of the atomic concentration of each element and (b) calculated bandgap depth profile for the PVA-CIGSSe film.


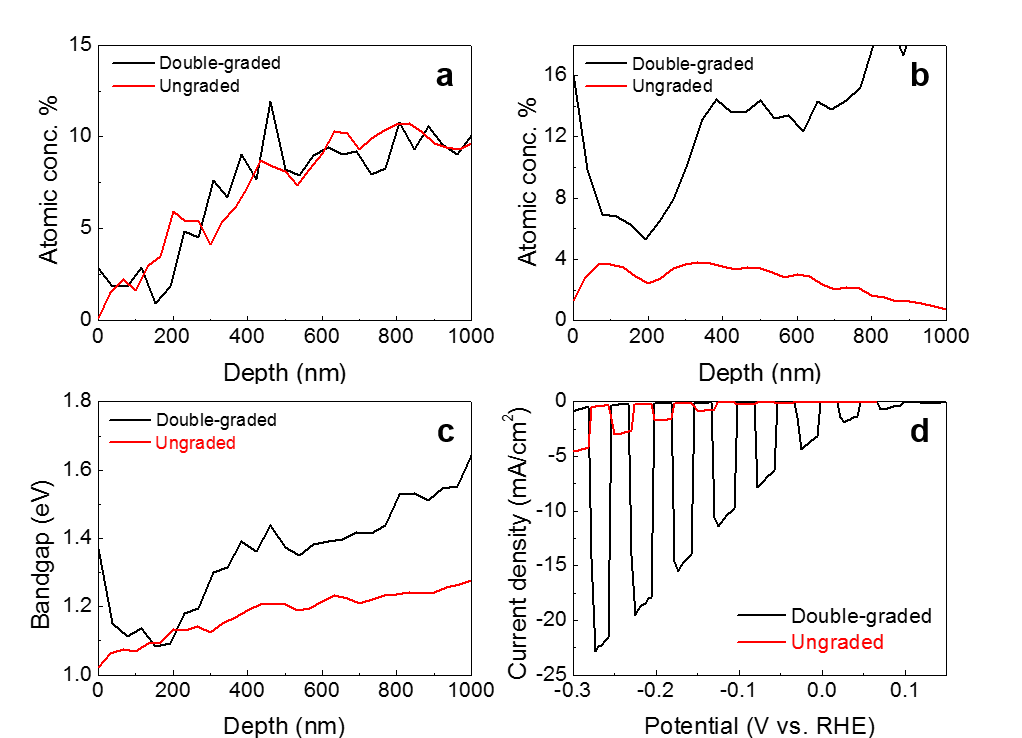


Figure S3. (a) Ga and (b) S atomic concentration and (c) bandgap profiles of the double-graded and the ungraded films according to the film depth. (d) I-V curves of the double-graded and the ungraded photocathodes. Despite similar Ga gradient feature, the ungraded film has weak bandgap gradient toward the substrate and absence of bandgap gradient toward the film surface due to uniformly distributed S throughout the film thickness. PEC activities of the ungraded film was also measured to be significantly low compared to those of the graded films (Figure S3d) implying strong correlation between PEC activity and bandgap gradient of the film.


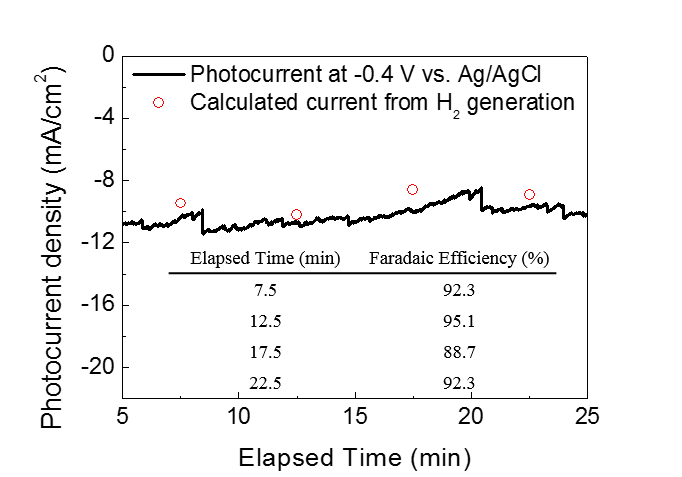


Figure S4. Chromoamperometry plot of PVA-CIGSSe photocathode during H_2_ generation quantification by a gas chromatography (GC). Red circles are the calculated current density values from the evolved H_2_ amount measured by GC. Inset table shows calculated Faradaic efficiency.
